# The prevalence and burden of four major chronic diseases in the Shanxi Province of Northern China

**DOI:** 10.3389/fpubh.2022.985192

**Published:** 2022-09-30

**Authors:** Lu He, Yuanyuan La, Yan Yan, Yuxiao Wang, Xi Cao, Yutong Cai, Sitian Li, Mengxia Qin, Qilong Feng

**Affiliations:** ^1^Department of Social Medicine, School of Public Health, Shanxi Medical University, Taiyuan, China; ^2^School of Social Development and Public Policy, Beijing Normal University, Beijing, China; ^3^Department of Health Economics, School of Management, Shanxi Medical University, Taiyuan, China; ^4^Key Laboratory of Cellular Physiology at Shanxi Medical University, Ministry of Education, Taiyuan, China

**Keywords:** chronic diseases, disease burden, disability-adjusted life years, attributable disease burden, coronary heart disease, diabetes, behavioral risk factors

## Abstract

**Background:**

Chronic non-communicable diseases constitute an important public health problem that is closely related to behavioral risk factors. The study examined the prevalence, burden, and behavioral risk factors relevant to four major chronic diseases in Shanxi Province, China. The results obtained could provide a basis for the formulation of chronic disease prevention and control strategies in north China.

**Methods:**

A multi-stage random sampling method was used to select 14,137 residents aged ≥15 years who completed a questionnaire survey and physical examination. The disease burden was evaluated using the disability-adjusted life years (DALY) index. The extent of disease burden attributable to smoking and drinking behavior was analyzed using counterfactual analysis.

**Results:**

The total DALYs due to the four major chronic diseases was 938,100. The years of life lost due to stroke accounted for 74.86%; the years of life lived with disabilities accounted for 54.0 and 68.1% of the total disease burden of coronary heart disease and diabetes. Coronary heart disease attributed to smoking (105,600) was the highest, followed by stroke (77,200), hypertension (6,000), and diabetes mellitus (5,900). Stroke attributed to drinking (30,700) was the highest followed by coronary heart disease (16,700) and diabetes (1,100). The disease burden caused by smoking and drinking was higher in men (164,000 and 40,700, respectively) than in women (30,700 and 7,300, respectively).

**Conclusion:**

There is a high prevalence and significant burden associated with major chronic diseases in Shanxi Province. Therefore, the need for the application of various interventions to control smoking and drinking (the major predisposing factors) should be applied to reduce this burden.

## Introduction

With social-economic development, acceleration of urbanization, and population aging, chronic non-communicable diseases have become a global public health issue that is associated with premature death and disability (1). According to the Global Burden of Disease (GBD) study conducted in 2017 (2), chronic diseases have become one of the leading causes of death worldwide. The top four leading non-communicable diseases of death are due to cardiovascular and cerebrovascular diseases (44%), cancer (22%), chronic respiratory diseases (9%), and diabetes mellitus (4%) (3). In recent years, China has faced great challenges in the field of chronic disease prevention and control. According to the National Health Commission of the People's Republic of China, more than 300 million patients have been diagnosed with chronic diseases, which accounted for 86.6% of the total causes of death.

The rapid increase in chronic disease morbidity and mortality has affected national health and poses an immense societal burden. In the most recent analysis of the GBD study, chronic diseases accounted for the majority (62%) of the total GBD, expressed as the disability-adjusted life years (DALY), representing an increase of 16% from 2007 to 2017 (4). The DALY caused by chronic diseases accounts for 82.75% of the total causes of death in China, and the DALY lost from the four major chronic diseases accounted for 59.06% of total chronic disease DALYs (5). The occurrence and development of chronic diseases are closely related to key lifestyle-based risk factors. The results of GBD 2019 showed that the top three attributable risk factors for deaths of women globally were high systolic blood pressure, poor diet, and hyperglycemia. For men, the risk factors were tobacco exposure (smoking, secondhand smoke, and the use of chewing tobacco), high systolic blood pressure, and poor dietary habits (6). The results of the surveillance survey of chronic diseases and their risk factors in China showed a high prevalence of bad lifestyle habits such as smoking, poor eating habits, and excessive drinking among Chinese residents (7), leading to a rise in the incidence of chronic diseases and a heavy disease burden. Therefore, analyzing the disease burden of major chronic diseases and behavioral risk factors is of great importance for promoting the development of preventive and treatment strategies.

Shanxi Province is located in north China, with a written history dating 3,000 years back, and is known as the “cradle of Chinese civilization”. The permanent resident population is about 34.8 million, and the proportion of people >60 years old is 18.92% (8). In contrast, the medical care of Shanxi Province is relatively poorly developed. According to the data from the China Health Statistical Yearbook, the total health expenditure per capita of Shanxi Province is 3,282 yuan, which is among the lowest five in China (9). Studies on the burden of non-communicable diseases in Chinese provinces and cities are ongoing. Ma et al. (10) estimated the death and disease burden of cardiovascular and cerebrovascular diseases in Beijing based on data from GBD 2016. Chen et al. (11) analyzed the DALY and economic burden of smoking in Hangzhou city in 2013. There are comparatively fewer studies on the disease burden of chronic diseases and risk factors in Shanxi Province in Northern China. Therefore, quantifying the disease burden to improve the prevention and control of major diseases in north China and inform public health policies is an urgent challenge.

In this study, we investigated the prevalence of chronic diseases among 14,137 residents of Shanxi Province enrolled using a multi-stage cluster sampling approach. According to the prevalence ranking, the four most prevailing chronic diseases were hypertension, diabetes mellitus, coronary heart diseases (CHD), and stroke. The disease burden caused by major chronic diseases, smoking and drinking- attributable disease burden, and cause of death monitoring data were evaluated.

## Materials and methods

### Data sources

Data on the cause of death were obtained from “The Chinese Cause of Death Surveillance Dataset 2017”. We used the death data of Central China, including the eight provinces of Shanxi, Jilin, Heilongjiang, Anhui, Jiangxi, Henan, Hubei, and Hunan. The Chinese cause of death surveillance system monitors a population of more than 300 million, with good provincial representation. The dataset is under strict quality control by eliminating data from some monitoring points that are considered to have serious underreporting and have the potential to affect the overall results. Data on chronic disease prevalence were obtained from the Shanxi Provincial Chronic Disease Current Conditions survey conducted from 2017 to 2019, which covered 11 cities in the Shanxi Province. Sex- and age-stratified population-based data were obtained from the sixth census data in the Shanxi Provincial Statistical Yearbook.

### Research methods

#### Sampling method

Eleven cities in Shanxi Province were used as sampling units in rural areas. A multi-stage random sampling method was used. Five districts (counties) were randomly selected in each city, two to three villages were randomly selected in each district (county), 50 households were selected in the selected villages according to the simple random sampling method, and two permanent residents (residents for at least 6 months) aged 15 years or older were selected in each household as study participants. In Taiyuan City, a total of 13 streets were selected by multi-stage random sampling method, and three to four communities were selected for each street. One hundred households were randomly selected from the selected communities, and two permanent residents (living or having lived for 6 months) aged 15 years or above were selected from each household as the study participants. A total of 15,000 questionnaires were distributed, and 14,137 valid questionnaires were returned, with a valid return rate of 94.25%.

#### Survey content

- Questionnaires: Self-designed questionnaires were used, and on-site surveys were administered by uniformly trained investigators. The content included general demographic characteristics, chronic disease prevalence (e.g., chronic disease types, family disease history, and medication compliance), and lifestyle factors (e.g., smoking, alcohol consumption, diet, and physical exercise). Chronic diseases were self-reported by residents diagnosed at a local medical institution at the county level or above. Smokers were defined as those who smoked more than one cigarette per day and smoked continuously or cumulatively for more than 6 months, and those who drank alcohol more than once a week, and drank continuously or cumulatively for more than 6 months, were defined as alcohol drinkers.
- Physical measurements: Height, weight, waist circumference, and blood pressure were measured by uniformly trained medical examiners, and blood glucose levels were tested. Patients with hypertension were defined as those with systolic blood pressure ≥140 mmHg and/or diastolic blood pressure ≥90 mmHg; or those with a previous history of hypertension who were currently taking anti-hypertensive medication and whose blood pressure had fallen below these standards. Patients with diabetes mellitus were defined as those with fasting blood glucose ≥7.0 mmol/L or blood glucose level ≥11.1 mmol/L after a 2 h oral glucose tolerance test, or with a previous history of diabetes mellitus and currently taking anti-diabetic medication.

### The DALY

To emphasize the influence of chronic disease caused early death on the life reducing, we applied YLL to evaluate the disease burden of chronic disease, including YLL and YLL rate. The age range of “premature death” was from 0 to >80 years old. The YLL rate was calculated from the 6th census data in the statistical yearbook of Shanxi Province. The calculation method was as follows:


(1) $$\begin{array}{l} YLL=N\times L \end{array}$$

N is the number of deaths for each age and sex group, and L is the value of life lost for each age group. We used the 2000–2017 life expectancy table from the World Health Organization's Burden of Disease Study to calculate the standard life expectancy for each age group (12).


(2) $$\begin{array}{l} YLLrate=\frac{YLL}{P}\times 1000 \end{array}$$

where P is the number of people in each age group.


(3) $$\begin{array}{l} YLD=\text{Pr }ev\times DW \end{array}$$

Where Prev is the number of patients with sequelae of a disease in a certain age group, and DW is the disability weight for that sequelae.

The weight of disability in this study was adopted from the GBD data, where the weight of hypertension and its complications was 0; the weight of diabetes mellitus was 0.033; the weight of stroke was 0.244 for the 15–60 years age group and 0.258 for the over 60 years age group and older, and the weight of CHD was 0.395 (13).


(4) $$\begin{array}{l} YLDrate=\frac{YLD}{P}\times 1000 \end{array}$$

P is the number of people in each age group.

The chronic disease burden attributable to lifestyle risk factors including smoking and drinking was calculated with reference to the RR values from the GBD 2017 study (2), with a theoretical minimum exposure level of 0 cigarettes/day and 0 g/day for smoking and drinking respectively. The risk attributable to smoking was calculated as:


(5) $$\begin{array}{l} PAF=\frac{P\;*\;\left(RR-1\right)}{P\;*\;\left(RR-1\right)+1} \end{array}$$

Where P is the prevalence of smoking and RR is the relative risk.

The risk of attributable to drinking was calculated as follows:


(6) $$\begin{array}{l} PAF=\frac{\overset{n}{\underset{i=1}{\sum }}P_{i}\left(RR_{i}-1\right)}{\overset{n}{\underset{i=1}{\sum }}P_{i}\left(RR_{i}-1\right)+1} \end{array}$$

Where P_i_ is prevalence of drinking at the category i, RR_i_ is the relative risk at level i, and n is the number of exposure levels.

The attributed disease burden due to smoking and drinking is given by:


(7) $$\begin{array}{l} AB_{x}=\underset{j}{\sum }B_{j}*PAF_{xj} \end{array}$$

Where AB_x_ is the disease burden attributable to smoking and drinking, B_j_ is the disease burden of disease j (YLL, YLD, and DALY), and PAF_xj_ is the population attribution score of risk factor x and disease j.

### Statistical analysis

We calculated the indicators of disease burden for the four major chronic diseases, including the YLL, YLD, DALY, YLL rate, YLD rate, and DALY rate. Data from surveillance of chronic diseases and their risk factors were entered using Epidata version 3.1 to create a database. Data were analyzed using SPSS version 24.0 for descriptive analysis of chronic disease prevalence. Counterfactual analysis was used to calculate the burden of disease attributable to smoking and alcohol consumption for the four major chronic diseases of interest in this study.

### Ethics statement

The studies involving human participants were reviewed and approved by Shanxi Medical University Ethics Committee. Written informed consent to participate in this study and publication of any potentially identifiable images or data included in this article was provided by the participants or their legal guardian/next of kin.

## Results

### Prevalence of major chronic diseases in Shanxi province

The top five chronic diseases in rural areas were hypertension (28.90%), diabetes mellitus (9.84%), rheumatoid arthritis (6.60%), CHD (3.31%), and chronic gastric/duodenal ulcer (2.84%); The top five chronic diseases in urban areas were hypertension (23.19%), diabetes mellitus (10.44%), CHD (4.75%), stroke (2.77%), and osteoporosis (2.26%). In this study, hypertension, diabetes mellitus, CHD, and stroke were selected as the main chronic diseases in residents >15 years old residing in Shanxi Province (Table 1).

**Table 1:** Prevalence of chronic diseases and their ranking.

**Types of chronic diseases**	**Frequency**	**Percent**	**Order**
**Rural**			
Hypertension	2,114	28.90	1
Diabetes	720	9.84	2
Rheumatoid arthritis	483	6.60	3
Coronary heart disease	242	3.31	4
Chronic gastritis and	208	2.84	5
gastroduodenal ulcer			
Liver cirrhosis	159	2.17	6
Stroke	139	1.90	7
COPD	106	1.45	8
Depression	100	1.37	9
Chronic renal failure	89	1.22	10
Cancer	46	0.63	11
**Urban**			
Hypertension	1,582	23.19	1
Diabetes	712	10.44	2
Coronary heart disease	324	4.75	3
Stroke	189	2.77	4
Osteoporosis	154	2.26	5
Cataracts	100	1.47	6
Chronic bronchitis	83	1.22	7
Mental disorder	43	0.63	8
Prostate hyperplasia	36	0.53	9
Cancer	30	0.44	10
Liver cirrhosis	24	0.35	11

### Analysis of disease burden of major chronic diseases in Shanxi province

**Figure 1:**
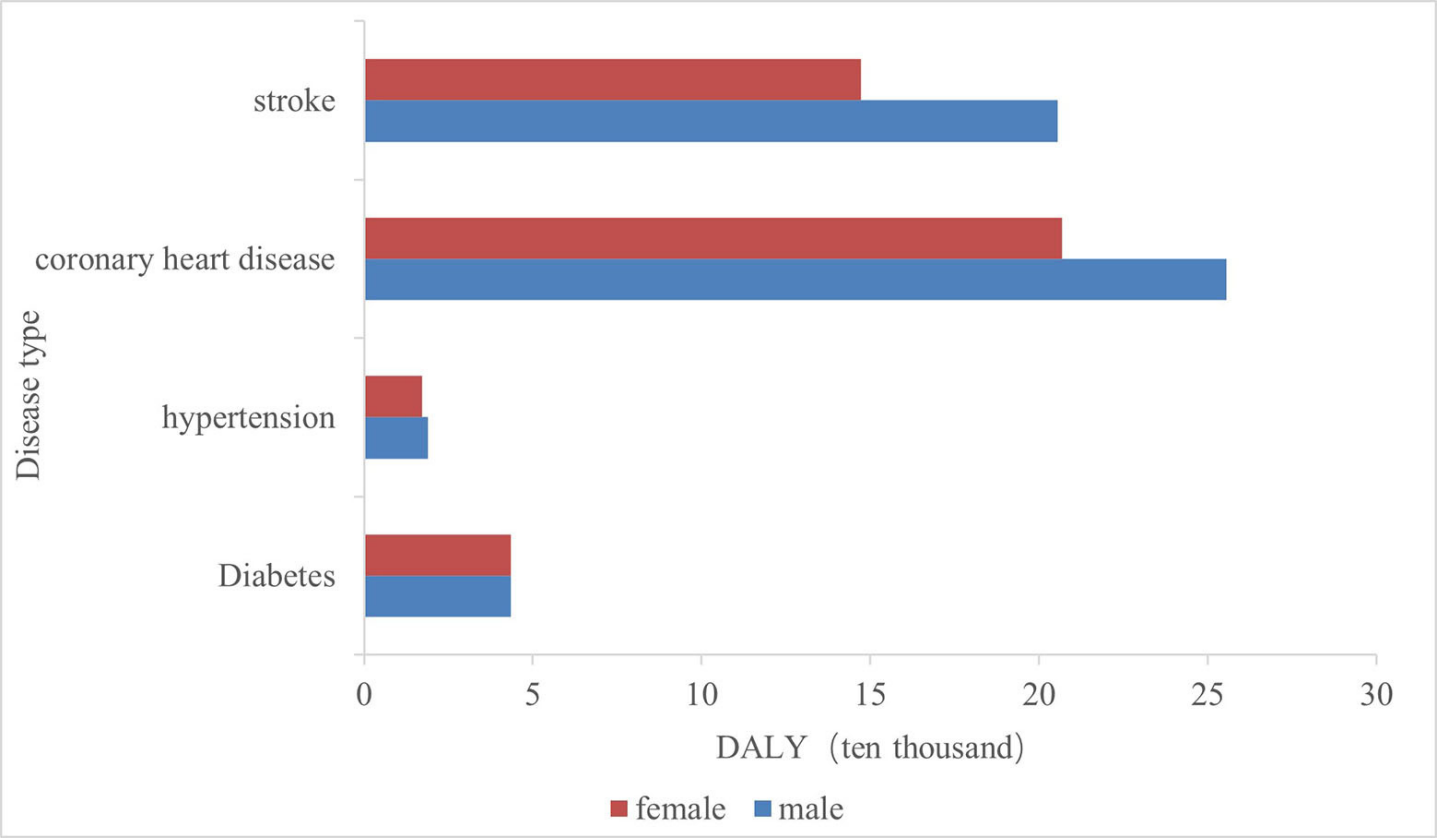
Distribution of the DALY by sex for major chronic diseases.

The four major chronic diseases in Shanxi Province caused a total of 938,100 person-years of DALY, in descending order: CHD, stroke, diabetes mellitus, and hypertension (Figure 1). All four chronic diseases caused a higher disease burden in men compared to women.

Diabetes mellitus and CHD had the highest DALY loss in the 45–59-year-old age group, stroke in the 60–69-year-old age group, and hypertension in the >80-year-old group (Table 2). The DALY rate for diabetes mellitus, hypertension, CHD, and stroke all increased with age, and peaked in the >80-year-old age group.

**Table 2:** Distribution of DALY in different age groups for major chronic diseases.

**Age (years)**	**Diabetes**		**Hypertension**		**Coronary heart disease**		**Stroke**	
	**DALY**	**DALY rate**	**DALY**	**DALY rate**	**DALY**	**DALY rate**	**DALY**	**DALY rate**
0–4	0.00	0.01	0.00	0.00	0.00	0.00	0.00	0.02
5–14	0.01	0.01	0.00	0.00	0.00	0.00	0.01	0.03
15–29	0.29	0.31	0.02	0.02	0.87	0.94	0.34	0.37
30–44	1.23	1.37	0.12	0.14	4.23	4.71	2.45	2.73
45–59	2.86	3.96	0.53	0.73	12.38	17.14	8.25	11.42
60–69	2.19	9.32	0.77	3.27	11.49	48.88	9.70	41.28
70–79	1.52	11.22	1.03	7.60	10.76	79.42	8.86	65.39
>80	0.60	14.57	1.15	27.95	6.49	157.28	5.65	137.05
Total	8.69	2.43	3.62	1.01	46.22	12.94	35.28	9.88

**Figure 2:**
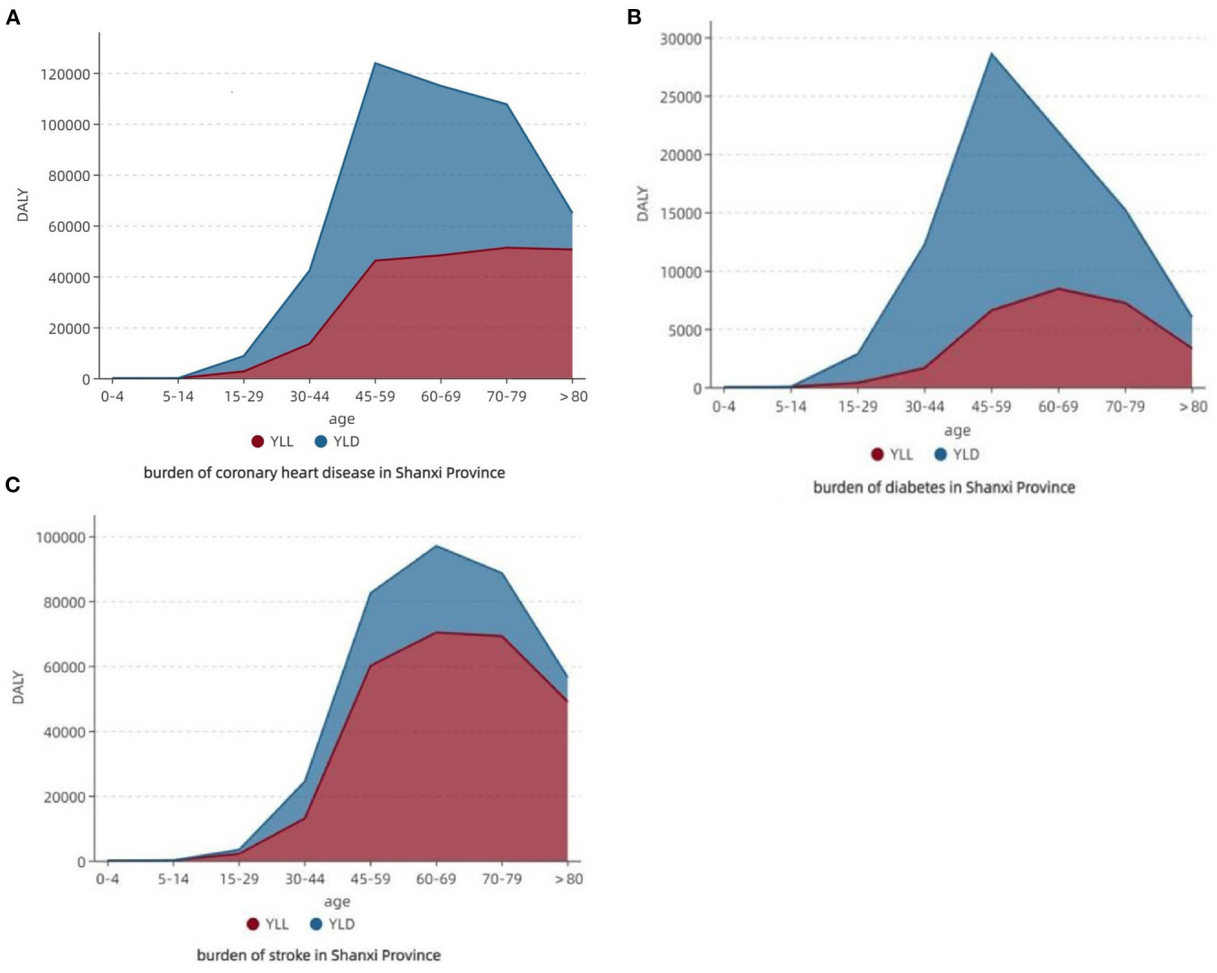
Disease burden and composition of three chronic diseases.

The composition of the disease burden of CHD was larger in YLD; the largest share of disease burden DALY was in the 45–59-year-old age group (26.78%), YLL in the 70–79-year-old age group (51,307.02 person-years), and YLD in the 45–59 years (77,552.00 person-years). The disease burden composition of diabetes mellitus was larger in YLD; DALY was the largest in the 45–59 age group (32.90%), YLL was the largest in the 60–69-year-old age group (8,438.38 person-years), and YLD was the largest in the 45–59-year-old age group (21,991.61 person-years). The disease burden composition of stroke was greater for YLL; the disease burden of DALY was greatest in the 60–69-year-old age group (27.50%), and both YLL and YLD were greatest in the 60–69-year-old age group, with 70,357.79 person-years and 26,652.88 person-years, respectively (Figure 2).

### Estimation of the disease burden attributable to smoking and drinking in Shanxi province

The population attributable fraction (PAF) of four chronic disease deaths due to smoking in Shanxi Province were, in descending order: CHD, stroke, hypertension, and diabetes mellitus. These were all higher in men than in women. Among them, the PAF of men with CHD, stroke, and hypertension gradually decreased with age, and the PAF was higher than 30% in the 30–60-year-old age group (Table 3).

**Table 3:** Attribution scores of smoking by sex and age group in the Shanxi Province (%).

**Age (years)**	**Coronary heart disease**		**Stroke**		**Diabetes**		**Hypertension**	
	**Man**	**Woman**	**Man**	**Woman**	**Man**	**Woman**	**Man**	**Woman**
30-	57.35	13.97	56.30	13.66	14.83	0.31	56.06	8.93
35-	50.99	9.24	50.03	9.05	13.29	0.23	49.85	5.89
40-	50.71	9.06	49.72	8.86	14.69	0.26	49.52	5.85
45-	49.26	9.47	48.24	9.26	15.65	0.31	48.12	6.21
50-	46.90	12.77	45.99	12.54	16.30	0.51	45.86	8.62
55-	44.69	12.01	43.80	11.75	17.05	0.56	43.65	8.18
60-	37.03	11.32	36.38	11.07	14.94	0.62	36.21	7.79
65-	30.33	9.84	29.63	9.68	13.30	0.64	29.63	6.82
70-	23.57	6.11	23.04	6.03	11.51	0.46	23.04	4.27
75-	18.74	7.18	18.38	7.06	10.56	0.67	18.19	5.09
>80	10.54	5.18	10.22	5.12	7.79	0.69	10.22	3.73
Total	41.33	10.72	40.44	10.51	14.39	0.45	40.29	7.11

The PAF of deaths from three chronic diseases due to drinking in Shanxi Province were, in descending order: stroke, CHD, and diabetes mellitus. The PAF for CHD and stroke was higher for men than for women. The PAF was the highest in men aged 40–45 years old for CHD and stroke, and in women aged 60–70 years old for stroke and diabetes mellitus, respectively (Table 4).

**Table 4:** Attribution scores of drinking by sex and age group in the Shanxi Province (%).

**Age (years)**	**Coronary heart disease**		**Stroke**		**Diabetes**	
	**Man**	**Woman**	**Man**	**Woman**	**Man**	**Woman**
30-	5.85	1.66	14.57	2.16	0.04	2.55
35-	5.11	1.34	15.15	3.51	1.37	1.86
40-	6.55	1.35	16.46	3.49	1.20	1.88
45-	6.00	1.14	15.66	2.84	1.25	1.60
50-	5.39	1.25	15.83	2.73	1.15	1.79
55-	5.76	1.50	15.71	2.97	0.48	2.21
60-	5.74	1.59	13.24	2.77	0.20	2.34
65-	5.43	1.53	13.19	3.64	0.17	2.14
70-	4.98	1.06	11.72	2.08	0.20	1.54
75-	5.10	1.44	9.32	3.51	0.72	2.01
>80	4.92	0.94	11.29	1.54	0.05	1.38
Total	5.61	1.35	14.30	2.97	0.56	1.94

The DALY for the four major chronic diseases attributable to smoking in Shanxi Province was 194,700 person-years. YLL was 110,100 person-years, accounting for 56.55%, and YLD was 84,600 person-years, accounting for 43.45%. Of the four chronic diseases, CHD had the highest disease burden attributable to smoking, with a DALY of 105,600 person-years, followed by stroke (77,200 person-years), hypertension (60,000 person-years), and diabetes mellitus (5,900 person-years). The attributable YLD was higher than the YLL for CHD and diabetes, and the attributable YLL was higher than the YLD for stroke.

**Figure 3:**
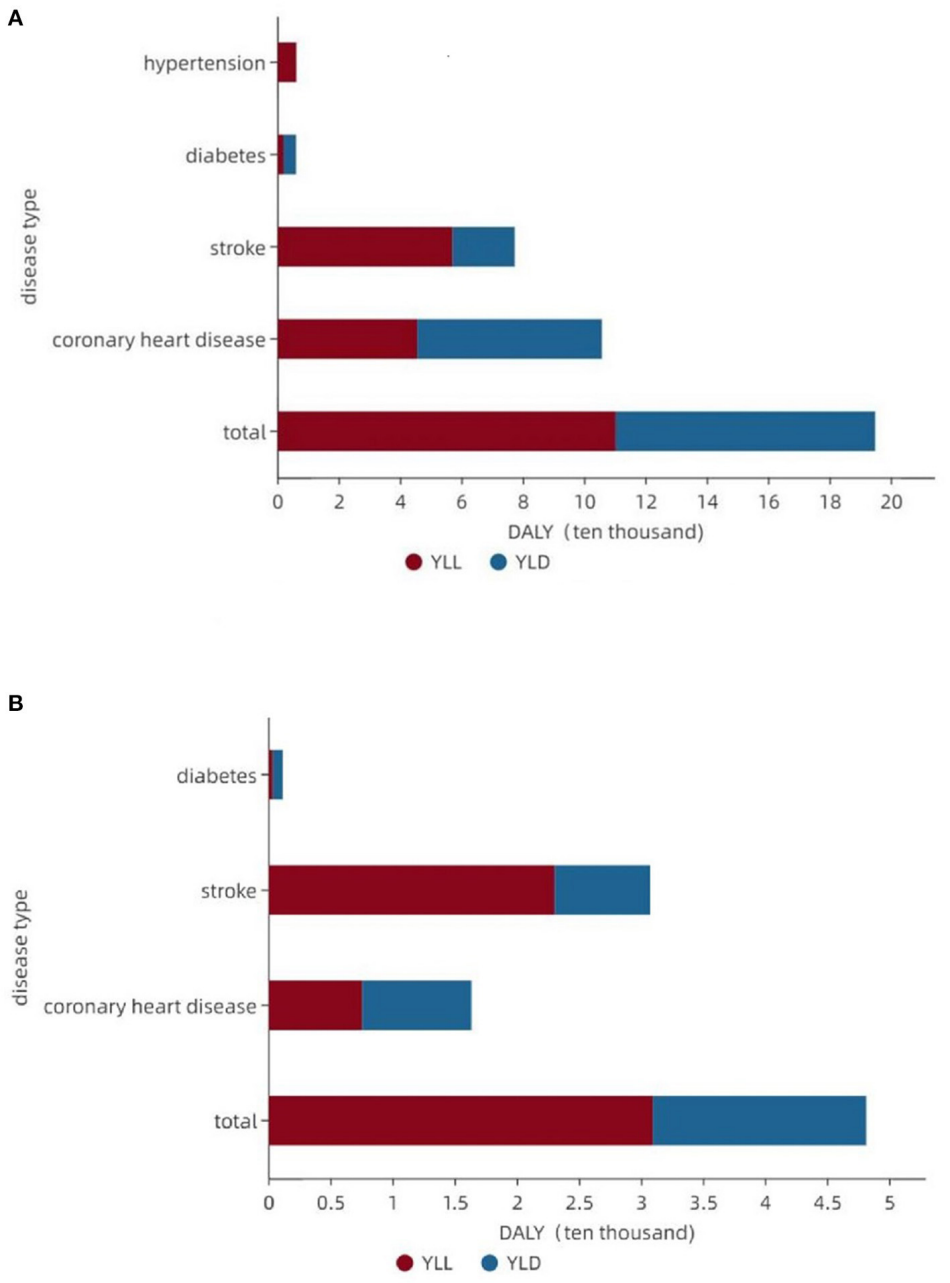
Composition of the disease burden attributable to smoking and drinking for major chronic diseases. **(A)** Major chronic disease burden attributable to smoking. **(B)** Major chronic disease burden attributable to drinking.

The DALY for the three main chronic diseases attributable to drinking was 48,100 person-years, YLL 30,900 person-years (64.24%), and YLD 17,200 person-years (35.76%). Stroke had the highest disease burden attributable to drinking, with a DALY of 30,700 person-years, followed by CHD (16,700 person-years), and diabetes mellitus (11,000 person-years). The attributable YLD was higher than the YLL for CHD and diabetes, and the attributable YLL was higher than the YLD for stroke (Figure 3).

In terms of sex, the smoking and drinking attributable YLL, YLD, and DALY were significantly higher in men than in women. In terms of age groups, the YLL, YLD, and DALY attributed to smoking showed an overall increasing trend in the 30–60 year old age group, and the disease burden attributed to smoking decreased significantly in the >60 year old age group; the YLD and DALY attributed to drinking showed a trend of an initial increase followed by a decrease with age, and the disease burden reached the highest in the >60 year old age group, and then decreased significantly (Table 5).

**Table 5:** Burden of major chronic diseases attributed to smoking and drinking in different sex and age groups in the Shanxi Province (10,000 person-years).

**Group**	**Smoking**			**Drinking**		
	**YLL**	**YLD**	**DALY**	**YLL**	**YLD**	**DALY**
**Sex**						
Man	9.32	7.07	16.4	2.66	1.41	4.07
Woman	1.69	1.39	3.07	0.42	0.31	0.73
**Age (years)**						
30-	0.22	0.37	0.59	0.03	0.04	0.07
35-	0.33	0.37	0.69	0.06	0.06	0.12
40-	0.63	0.91	1.54	0.14	0.17	0.31
45-	1.16	0.87	2.03	0.25	0.14	0.4
50-	1.59	1.17	2.76	0.35	0.2	0.54
55-	1.29	1.51	2.8	0.31	0.26	0.57
60-	1.64	1.27	2.91	0.41	0.27	0.68
65-	1.47	0.8	2.27	0.45	0.2	0.65
70-	1.01	0.63	1.64	0.35	0.18	0.52
75-	0.87	0.38	1.25	0.32	0.11	0.43
>80	0.81	0.18	0.99	0.42	0.1	0.51
Total	11.01	8.46	19.47	3.09	1.72	4.81

## Discussion

This is the first study to quantitatively estimate the disease burden of major chronic diseases in Shanxi Province. Here, we used DALY as a metric and evaluated the disease burden of major chronic diseases caused by two common risk factors, i.e., smoking and drinking. The latest national health services survey showed that the most common chronic diseases were hypertension, diabetes mellitus, and CHD (9).

In this study, the prevalence of these four diseases was high. Owing to the differences in population, age structure, and lifestyle habits across different provinces of China, there are geographical differences in the prevalence of major chronic diseases. According to the results of the previous nationwide survey, the prevalence of diabetes mellitus and hypertension is higher in the northern region than in the southern region of China (14–16). The result of this study showed a lower prevalence of diabetes mellitus in the urban area than in a province in northeast China (20.21%) (17), but higher than in a province in southeastern coastal (9.4%) (18). The prevalence of hypertension in the rural area was higher than that of Jiangxi Province (24.04%) (19), lower than that of Ningxia Hui Autonomous Region (20) in line with the characteristics of “high in the north and low in the south”. There is still a high prevalence of CHD and stroke in China, with a steady increase from 1990 to 2017, which is still ongoing (21). In recent overviews, the incidence and mortality rates of stroke in China appear to be the highest in the world (22). Compared to a study conducted in rural areas in Shanxi Province in 2017 (23), the prevalence of all four diseases increased. Therefore, in the prevention and control of chronic diseases, Shanxi Province should take them as the major focus and establish a long-term investment mechanism to maximize the effectiveness of prevention and control.

We found that CHD was the most significant contributor to DALY among the residents of Shanxi Province. In China, the DALY rate for cardiovascular diseases (CVD) has declined in every province from 1990 to 2016, but the northern provinces had the highest age-standardized DALY rate for ischemic stroke (24). In this study, the DALY rate was higher than those of Sichuan province (25) and Tianjin city (26), especially the YLD rate. The main burden of CHD in Shanxi Province was therefore the loss of life caused by disabling life-threatening conditions. The difference between provinces is partly due to the gaps in cardiovascular care (27, 28), demonstrating the importance of increasing investment in the prevention and treatment of CHD in Shanxi Province. Stroke was second only to CHD as a cause of disease burden in Shanxi province. As the third leading cause of death in China, stroke has a higher disease burden than the global average, which will continue to increase in the next decade (29). Early death is the leading cause of stroke disease burden in China (30). YLL accounts for 76% of the total burden of disease in this study, owing to the many rural areas in Shanxi Province and relatively scarce medical resources. Thus, the YLL was higher than YLD because of delayed treatment after stroke. The capacity building of stroke treatment should be strengthened in the future, and the capacity of stroke diagnosis and treatment at the grassroots level should be improved.

The impact of smoking and excessive alcohol use on adverse cardiac and cerebrovascular outcomes is well-established. Our study also discovered that CHD had the highest disease burden attributable to smoking, with a DALY of 105,600 person-years. Among smoking-related deaths, CVD accounts for approximately one-third of cases worldwide (31). Tobacco smoking, both active and passive (i.e., secondhand smoke), increases the incidence of all phases of atherosclerosis, from endothelial dysfunction to various types of CVD (32, 33). Even smoking a single cigarette daily increases the risk of developing CHD and stroke. Currently, smoking is not yet completely banned in public places and indoor workplaces in the Shanxi Province, which still has a high smoking rate. Beijing has effectively reduced the number of smokers and decreased the hospital admission of CVD by adopting the strictest tobacco control policy implemented in China to date (34). Therefore, it is necessary to take more stringent measures to reduce the CHD burden caused by the use of tobacco products.

Notably, the burden of disease attributable to alcohol consumption was greater for stroke than for CHD, implying that moderate alcohol consumption may have a protective effect against CVD. The relationship between alcohol consumption and CVD is controversial (35). The relationship between alcohol intake and CVD risk is mostly dose-dependent, i.e., the greater the amount of alcohol consumed, the greater the relative increase in disease risk. However, in several meta-analyses, regular alcohol consumption in small amounts was found to have a protective effect against CHD and ischemic stroke (36, 37). A study conducted in Argentina also revealed that drinking was estimated to save 85,772 DALY from CHD, but was responsible for 52,171 lost from stroke (38).

In addition, the DALY rate showed an increasing trend in the 30–60 age group and began to gradually decline above the age of 60 years. This is related to the higher prevalence of smoking and alcohol consumption in the 30–60 years age group, which began to decline gradually after the age of 60 years. Aging is perhaps the most important risk factor affecting cardiovascular homeostasis (39). The disease burden attributable to smoking and alcohol consumption is significantly higher in men than in women, consistent with a study conducted in China (40). Thus, sex and age should be considered in the prevention of chronic diseases, focusing on screening and intervention of important factors. The occurrence of cardiovascular and cerebrovascular disease deaths due to smoking and alcohol consumption is a process with long-term effects, and interventions in smoking and alcohol consumption from adolescence have important potential benefits in preventing death in middle and old age (41).

Our study had a few limitations. First, the mortality data were obtained from the central regional data of the Chinese cause of death surveillance data set, which might have resulted in underestimated uncertainties for the DALY. Second, this study used relevant parameters from the GBD studies, such as disability weights and relative risk of disease, which may not be fully applicable to Shanxi Province, and may introduced some bias to the study results. However, the use of common parameters is beneficial to the comparison of results between different regions. Thirdly, chronic diseases were identified based on self-report, which may be affected by measurement errors or lack of accuracy. However, the literature shows that self-reported measures of chronic diseases are widely used in large population-based studies and show reasonable accuracy.

The situation of chronic disease prevalence in Shanxi Province remains serious, with a high prevalence of hypertension and diabetes mellitus. The disease burden caused by major chronic diseases is high, and reducing smoking and drinking behaviors can help reduce the disease burden caused by premature death due to hypertension, diabetes mellitus, CHD, and stroke. The relevant departments need to focus on the prevention and control of major chronic diseases, pay attention to the changes in the DALY loss by sex and age group, develop targeted prevention and control strategies, strengthen the health education of the population, and promote smoking cessation and alcohol restriction to effectively reduce the level of population mortality and increase life expectancy.

## Data availability statement

The raw data supporting the conclusions of this article will be made available by the authors, without undue reservation.

## Ethics statement

The studies involving human participants were reviewed and approved by Shanxi Medical University Ethics Committee. Written informed consent to participate in this study and publication of any potentially identifiable images or data included in this article was provided by the participants or their legal guardian/next of kin.

## Author contributions

LH, QF, and YL conceived the idea. YW, YY, and YL participated in data collection and statistical analysis. YC, XC, SL, and MQ gave many valuable comments on the draft and polished it. All authors have read and approved the manuscript.

## Funding

Funding for this study was received from the Population Strategy Project from the Health Commission of Shanxi Province (RK05) and the Natural Science Foundation of Shanxi Province, 201901D111195 (QF).

## Conflict of interest

The authors declare that the research was conducted in the absence of any commercial or financial relationships that could be construed as a potential conflict of interest.

## Publisher's note

All claims expressed in this article are solely those of the authors and do not necessarily represent those of their affiliated organizations, or those of the publisher, the editors and the reviewers. Any product that may be evaluated in this article, or claim that may be made by its manufacturer, is not guaranteed or endorsed by the publisher.

## References

[1] GBD 2015 Mortality and Causes of Death Collaborators. Global, regional, and national life expectancy, all-cause mortality, and cause-specific mortality for 249 causes of death, 1980-2015: a systematic analysis for the Global Burden of Disease Study 2015. Lancet. (2016) 388:1459–544. 10.1016/S0140-6736(16)31012-1 27733281 PMC5388903

[2] GBD 2017 Causes of Death Collaborators. Global, regional, and national age-sex-specific mortality for 282 causes of death in 195 countries and territories, 1980-2017: a systematic analysis for the Global Burden of Disease Study 2017. Lancet. (2018) 392:1736–88. 10.1016/S0140-6736(18)32203-7 30496103 PMC6227606

[3] World Health Organization. World Health Statistics 2016: Monitoring Health for the SDGs, Sustainable Development Goals. Geneva: World Health Organization (2016).

[4] GBD 2017 DALYs and HALE Collaborators. Global, regional, and national disability-adjusted life-years (DALYs) for 359 diseases and injuries and healthy life expectancy (HALE) for 195 countries and territories, 1990-2017: a systematic analysis for the Global Burden of Disease Study 2017. Lancet. (2018) 392:1859–922. 10.1016/S0140-6736(18)32335-3 30415748 PMC6252083

[5] Yin P Qi JL Liu YN Liu J Li J Zeng X. Burden of disease in the Chinese population from 2005 to 2017. Chin Circ J. (2019) 34:1145–54. 10.1016/S0140-6736(19)30427-1 31474069

[6] GBD 2019 Risk Factors Collaborators. Global burden of 87 risk factors in 204 countries and territories, 1990-2019: a systematic analysis for the Global Burden of Disease Study 2019. Lancet. (2020) 396:1223–49. 10.1016/S0140-6736(20)30752-2 33069327 PMC7566194

[7] Chinese Center for Disease Control and Prevention. China Chronic Disease and Risk Factor Surveillance. Beijing: Military Medical Science Press (2016).

[8] National Bureau of Statistics. Bulletin of the Seventh National Population Census (No.5) (2021). Available online at: http://www.stats.gov.cn/tjsj/tjgb/rkpcgb/qgrkpcgb/202106/t20210628_1818824.html (accessed June 18, 2022).

[9] National Health Commission of the People's Republic of China. China Health Statistical Yearbook. Beijing: Peking Union Medical College Press (2021).

[10] Ma AJ Zhou MG Zeng XY Dong Z. Current status and changes of disease burden of cardio-cerebrovascular diseases in 1990 and 2016 for Beijing people. Zhonghua Xin Xue Guan Bing Za Zhi. (2020) 48:244–9. 10.3760/cma.j.cn112148-20190403-00164 32234183

[11] Chen JF Tang J Li JT Li L. An analysis on the disease burden of smoking in Hangzhou City. J Prevent Med. (2016) 28: 226–9. 10.19485/j.cnki.issn1007-0931.2016.03.00333441760

[12] World Health Organization. WHO Methods and Data Sources for Global Burden of Disease Estimates 2000-2017. World Health Organization (2019).

[13] Murray CJ Lopez AD. Quantifying disability: data, methods and results. Bull World Health Organ. (1994) 72:481–94. 8062403 PMC2486704

[14] Yang J Yu W Zhou Q Mahapatra T Li Y Zhang X. Burden and correlates of non-communicable-diseases among rural residents: a cross-sectional study in Hebei, China. BMC Public Health. (2015) 15:571. 10.1186/s12889-015-1916-x 26088558 PMC4473846

[15] Zhou M Astell-Burt T Bi Y Feng X Jiang Y Li Y. Geographical variation in diabetes prevalence and detection in china: multilevel spatial analysis of 98,058 adults. Diabetes Care. (2015) 38:72–81. 10.2337/dc14-1100 25352654 PMC4392931

[16] Li Y Wang L Feng X Zhang M Huang Z Deng Q. Geographical variations in hypertension prevalence, awareness, treatment and control in China: findings from a nationwide and provincially representative survey. J Hypertens. (2018) 36:178–87. 10.1097/HJH.0000000000001531 29210864

[17] Zhang FQ Tian YM Jing L Yan H Li SB Shi L. Morbidity and influence factors of diabetes mellitus among urban residents (≥40 years old) in Liaoning Province. Chin J Preven Control Chronic Dis. (2020) 28:823–7. 10.16386/j.cjpccd.issn.1004-6194.2020.11.006

[18] Zhang YQ Su J Lv SR Pan XQ Tao R Zhang FY. Analysis on diabetes awareness and its influential factors of urban and rural residents in Jiangsu Province. Chin J Health Educ. (2013) 29:891–3. 10.16168/j.cnki.issn.1002-9982.2013.10.030

[19] Huang X Cai H. Investigation on prevalence of hypertension and the risk factors among urban and rural residents in Jiangxi province. Chin J Hyperten. (2017) 25:169–75. 10.16439/j.cnki.1673-7245.2017.02.010

[20] Wang P Liu L. Prevalence and influence factors of chronic diseases among rural inhabitants in Ningxia Hui Autonomous Region. Chin J Public Health. (2014) 30:1516–20. 10.11847/zgggws2014-30-12-07

[21] Cao XX Xu CJ Hou YB Wang Y Pan N Xu FS. The epidemic trend and prediction of chronic diseases with high incidence in China from 1990 to 2025. Chin J Prev Contr Chron Dis. (2020) 28:14–9. 10.16386/j.cjpccd.issn.1004-6194.2020.01.004

[22] Thrift AG Thayabaranathan T Howard G Howard VJ Rothwell PM Feigin VL. Global stroke statistics. Int J Stroke. (2017) 12:13–32. 10.1177/1747493016676285 27794138

[23] Cao Q. Risk Factors Analysis of Common Chronic Disease and Establishment of a Risk Factors Appraisal Model—Taking Hypertension and Diabetes for Example. Taiyuan: Shanxi Medical University (2018).

[24] Liu S Li Y Zeng X Wang H Yin P Wang L. Burden of cardiovascular diseases in China, 1990-2016: findings From the 2016 Global Burden of Disease Study. JAMA Cardiol. (2019) 4:342–52. 10.1001/jamacardio.2019.0295 30865215 PMC6484795

[25] Wen XY Pan JP Duan ZQ Zeng ZY Chen Y Chen L. The burden of cardiovascular and cerebrovascular diseases in Sichuan Province from 2015 to 2017. Chin J Prevent Control Chron Dis. (2019) 29:14–7. 10.16386/j.cjpccd.issn.1004-6194.2021.01.003

[26] Liu M Zhou M Liu S Zeng X Zhang H Xu Z. The analysis on the burden of cardiovascular diseases in 1990 and 2015 of Tianjin. Chin J Prev Contr Chron Dis. (2018) 26:421–5. 10.16386/j.cjpccd.issn.1004-6194.2018.06.006

[27] Li Z Wang C Zhao X Liu L Wang C Li H. Substantial progress yet significant opportunity for improvement in stroke care in China. Stroke. (2016) 47:2843–9. 10.1161/STROKEAHA.116.014143 27758941

[28] Zhao D Liu J Wang M Zhang X Zhou M. Epidemiology of cardiovascular disease in China: current features and implications. Nat Rev Cardiol. (2019) 16:203–12. 10.1038/s41569-018-0119-4 30467329

[29] Yu XH Gao S Jia HH Jiang HR Qiang BYZ Shang PP. Study on the burden of Stroke in China and the World in 1999, 2009 and 2019. Chin Health Econ. (2021) 40:58–61. Available online at: https://kns.cnki.net/kcms/detail/detail.aspx?dbcode=CJFD&dbname=CJFDLAST2021&filename=WEIJ202106016&uniplatform=NZKPT&v=XQwDXiypy0Gxd7zaZzVJwoFCZoG8XCQwGVci8IfkJRFJniC-DUkjLX7hn8ovztBS

[30] China Stroke Study Collaboration. Stroke in China: advances and challenges in epidemiology, prevention, and management. Lancet Neurol. (2019) 18:394–405. 10.1016/S1474-4422(18)30500-3 30878104

[31] World Health Organization. The Tobacco Atlas (2019). Available online at: http://www.tobaccoatlas.org/ (accessed December 12, 2021).

[32] Ambrose JA Barua RS. The pathophysiology of cigarette smoking and cardiovascular disease: an update. J Am Coll Cardiol. (2004) 43:1731–7. 10.1016/j.jacc.2003.12.047 15145091

[33] Barua RS Ambrose JA. Mechanisms of coronary thrombosis in cigarette smoke exposure. Arterioscler Thromb Vasc Biol. (2013) 33:1460–7. 10.1161/ATVBAHA.112.300154 23685556

[34] Wu Y Wang Z Zheng Y Wang M Wang S Wang J. The impact of comprehensive tobacco control policies on cardiovascular diseases in Beijing, China. Addiction. (2021) 116:2175–84. 10.1111/add.15406 33404152

[35] Rehm J Gmel GE Sr. Gmel G Hasan O Imtiaz S Popova S. The relationship between different dimensions of alcohol use and the burden of disease-an update. Addiction. (2017) 112:968–1001. 10.1111/add.13757 28220587 PMC5434904

[36] Yoon SJ Jung JG Lee S Kim JS Ahn SK Shin ES. The protective effect of alcohol consumption on the incidence of cardiovascular diseases: is it real? A systematic review and meta-analysis of studies conducted in community settings. BMC Public Health. (2020) 20:90. 10.1186/s12889-019-7820-z 31964375 PMC6971904

[37] Bell S Daskalopoulou M Rapsomaniki E George J Britton A Bobak M. Association between clinically recorded alcohol consumption and initial presentation of 12 cardiovascular diseases: population based cohort study using linked health records. BMJ. (2017) 356:j909. 10.1136/bmj.j909 28331015 PMC5594422

[38] Bardach AE Caporale JE Rubinstein AL Danaei G. Impact of level and patterns of alcohol drinking on coronary heart disease and stroke burden in Argentina. PLoS ONE. (2017) 12:e0173704. 10.1371/journal.pone.0173704 28282416 PMC5345854

[39] Kovacic JC Moreno P Hachinski V Nabel EG Fuster V. Cellular senescence, vascular disease, and aging: part 1 of a 2-part review. Circulation. (2011) 123:1650–60. 10.1161/CIRCULATIONAHA.110.007021 21502583

[40] Wen H Xie C Wang F Wu Y Yu C. Trends in disease burden attributable to Tobacco in China, 1990–2017: Findings from the global burden of disease study 2017. Front Public Health. (2020) 8:237. 10.3389/fpubh.2020.00237 32766191 PMC7381278

[41] Wang M Luo X Xu S Liu W Ding F Zhang X. Trends in smoking prevalence and implication for chronic diseases in China: serial national cross-sectional surveys from 2003 to 2013. Lancet Respir Med. (2019) 7:35–45. 10.1016/S2213-2600(18)30432-6 30482646

